# Elderly at risk in care transitions When discharge summaries are poorly transferred and used –a descriptive study

**DOI:** 10.1186/s12913-018-3581-0

**Published:** 2018-10-11

**Authors:** Gabriella Caleres, Åsa Bondesson, Patrik Midlöv, Sara Modig

**Affiliations:** 1 Department of Clinical Sciences in Malmö/Family Medicine, Center for Primary Health Care Research, Lund University, Box 50332, SE-20213 Malmö, Sweden; 2 Department of Medicines Management and Informatics in Skåne County, Malmö, Sweden

**Keywords:** Elderly, Discharge summary, Primary care, Information transfer

## Abstract

**Background:**

Discharge summary with medication report effectively counteracts drug-related problems among elderly patients due to insufficient information transfer in care transitions. However, this requires optimal transfer and use of the discharge summaries. This study aimed to examine information transfer with discharge summaries from hospital to primary care.

**Methods:**

A descriptive study with data consisting of discharge summaries of 115 patients, 75 years or older, using five or more drugs, collected during one week from 28 different hospital wards in Skåne county, Sweden. Two weeks after discharge, information transfer was examined via review of primary care medical records. It was noted whether the discharge summary was received (i.e. scanned to the primary care medical records), if the medication list was updated with drug changes and if a patient chart entry regarding medication or its follow-up was made in the primary care medical records. An electronic survey, which was sent to 151 primary care units in Skåne county, was used to examine experiences of the information transfer.

**Results:**

Out of 115 discharge summaries, 47 (41%) were found in the primary care medical records. Patient chart entries regarding medication or its follow-up were seen in 53 (46%) cases. Drug changes during hospitalisation were seen in 51 out of 76 patients without multidose drug dispensing. In 16 (31%) out of these cases, medication lists were updated in primary care medical records.

In the electronic survey, 22 (21%) out of the 107 responding primary care units reported the discharge summary was *often* received on the day of discharge, while 71 (66%) respondents indicated the discharge summary was *always*/*often* received but later. Medication list updates and patient chart entries in the primary care medical records were *always/often* done upon receipt of the discharge summary according to 61 (57%) respondents.

**Conclusion:**

The transfer of information was often deficient and the discharge summaries were insufficiently used. Many discharge summaries were lost, an insufficient proportion of medication lists were updated and patient chart entries were often lacking. These findings may increase the risk of medication errors and drug-related problems for elderly in care transitions.

## Background

In Sweden, separate hospital and primary care medical records and thus separate medication lists are common. Hence, adequate information transfer in care transitions is important but is often insufficient [1–3], which increases the risk of medication errors [4].

Elderly people are at particular risk of re-admission and adverse outcomes after hospital discharge [5, 6]. Polypharmacy is common among the elderly [7, 8]. The risk of adverse drug reactions consequently increases [8, 9], which along with medication errors contributes to increased illnesses and high health care costs [10]. However, many adverse drug reactions are preventable according to both Swedish and American studies [8, 9, 11, 12]. Medication reconciliation and discharge summaries with medication reports reduce medication errors and health care consumption [7, 13–15]. Well-functioning routines for medical information transfer and follow-up are also essential in care transitions [7, 8], especially for the elderly where interventions to improve care transitions seem beneficial [16]. Furthermore, adequate follow-up in primary care requires correct discharge information [1]. The discharge summary must also fulfil the requirements of the general practitioners (GPs). In some previous European studies, the GPs primarily wanted immediately given and accurate information on drug changes [1, 17].

The advantages of discharge summaries with medication reports are well-known [15, 16], but the transfer of such summaries to primary care has been less explored. If the discharge summary is not properly transferred to and used in primary care, then the benefits may be lost. Increased knowledge of transfer and use of information may help improve care transitions, particularly for elderly patients who are at high risk of medication errors and adverse drug reactions.

The aim of this study was to assess the transfer of discharge summaries with medication reports from hospital to primary care both directly and through exploring primary care experiences, as well as the use of the information, i.e. the updating of medication lists in primary care electronic medical records after drug changes and making a patient chart entry regarding medication or its follow-up.

## Methods

### Setting

The study was conducted in Skåne county in southern Sweden where **1.3 million** (13%) of the Swedish population lives [18]. This region has ten hospitals of varying sizes and just over 150 primary care units. Both the hospitals and primary care units have separate electronic medical records. Current local guidelines in Skåne county state that the discharge summary should contain a medication report, which summarizes which changes in medication were made and why, as well as a medication list. The existing guidelines also state that the discharge summary should be sent to the next caregiver (i.e. in general the primary care provider but also municipal care) on the day the patient is discharged [19]. The discharge summary also contains brief information about the cause of hospitalisation, what happened during the hospital stay and any plans after discharge. The discharge summary is partly electronically generated (data elements such as the medication list, admission and discharge dates and patient name are automatically derived from the electronic medical records), while information such as what happened during the hospital stay and the medication report is written by a physician. The discharge summary is printed from the hospital electronic medical records and sent by post to primary care where it is scanned to the primary care electronic medical records. This document is also given to the patient and, if applicable, nurses in municipality care. Information transfer also occurs via a medical case history; a more detailed document on the hospital stay without a medication list or report that is intended only for the next caregiver.

### Study design

This descriptive study was a part of a Swedish regional quality improvement project on routines for and content of the discharge summary [20], mainly for patients with five or more drugs. The first part of the study was designed to study the receipt of discharge summaries done by primary care units, and updating of the medication information by answering the following questions:

- Is the discharge summary transferred from the hospital to the primary care electronic medical records?
- Is the medication list in the primary care electronic medical records updated after drug changes during hospitalisation?
- Is a patient chart entry regarding medication or medication-related follow-up made in the primary care electronic medical records?

Transfer was confirmed by finding the discharge summary in the primary care medical records. Information on any drug changes was derived from the discharge summaries as well as other hospital discharge documents such as the medical case history, referrals and so forth. Making a patient chart entry regarding medication or its follow-up is important since it signals an active stance being taken regarding the discharge summary with medication report.

Primary care electronic medical records were reviewed two weeks after hospital discharge in order to answer questions (a), (b) and (c) listed above, representing the descriptive variables; receipt of a discharge summary with medication list and notation of adjustments to the medication as well as any related follow-up.

The other part of the study was designed to assess primary care perspectives of the current processes of information transfer with discharge summaries as recorded by an electronic survey conducted in September–November 2015. A questionnaire was developed by two of the authors (ÅB and SM) in association with fellow pharmacists as a part of an overall regional quality improvement project. Questions concerned respondents’ opinions about the transfer of the discharge summaries, possible current routines and actual use as well as views on the content of the summaries. Questions included a Likert scale and free-text space to leave optional extra comments. Data from the Likert scale were quantified. The free-text comments were analysed according to summative content analysis of the manifest data [21], with the content grouped into categories that emerged during the analysis.

The survey results have been reported, without analyses, in a previous regional report. In this study, the answers were analysed and compared with the results of the first part of the study.

### Study sample

**Fig. 1:**
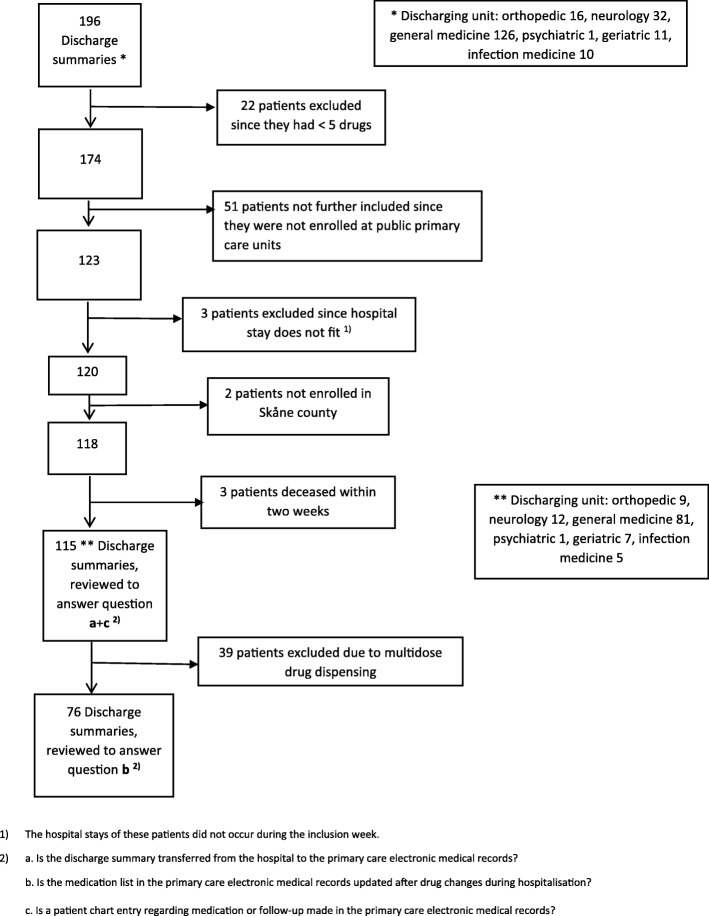
Inclusion flowchart

For one week in March 2015, all discharge summaries from 28 different hospital wards were included. Inclusion criteria were patients aged 75 years or older with five or more drugs that were enrolled at public primary care units (approximately 60% of inhabitants) (Fig. 1). Exclusion criteria were patients who died before the primary care medical record review was performed. Since the medication list in primary care medical records is commonly not used for patients with multidose drug dispensing, these patients were excluded from the research question (b) regarding updated medication lists. Multidose drug dispensing means machine-dispensed disposable sachets in which medications are packaged according to the time of administration [22].

The electronic survey was sent to all the heads of primary care units in the region by the department of Medicines Management and Informatics in Skåne County. The primary care units were recommended to answer the survey in consultation with the chief physician who holds the overall medical responsibility since the head of the primary care unit is seldom a physician.

### Statistical methods

Time span and number of wards was based on the number of discharge summaries that were produced in the preceding year. No differences in results were expected due to the excluded discharge summaries. To compensate for the expected loss due to enrolment at private primary care units, the number of wards was increased. We obtained a convenience sample of 120 discharge summaries, which was estimated to give enough data for descriptive analyses even after loss of those discharge summaries, which were not received by primary care or excluded due to the reasons listed above.

IBM SPSS Statistics 22.0 was used to process data, primarily for descriptive analyses. Categorical variables were compared using chi2 test.

### Ethical considerations

The ethical committee in Lund decided that there was no need for an ethics review regarding the descriptive part of the study (reference number 2015/858). The heads of the primary care units gave written approval for medical record reviewing. An approval for medical record reviewing was also obtained within the overall quality improvement project. It is not possible to identify any individual patient whose medical records was reviewed.

The survey was part of a quality improvement project within the regional health care organisation. The use of the survey results for research was approved by the head of the health care organisation. Since the survey was part of a quality improvement project there was no written or verbal participant consent but participation was voluntary. No patients were included and the answers were analysed without risk of identifying any primary care centre nor individual respondent.

## Results

Discharge summaries from 115 patients were included (Fig. 1). The average age for all patients was 85.5 years: 86.4 years for women and 84.6 years for men. A total of 61% of the patients (70/115) were women.

### Information transfer to primary care

Out of all discharge summaries, 47 (41%) were transferred to the primary care medical records. Discharge summaries were received to varying degrees from the various health care units: General medicine (41/81), Neurology (3/12) and Infection medicine units (3/5). No discharge summaries were received from the Orthopedic, Psychiatric or Geriatric units.

Drug changes during hospitalisation were seen in 51 out of the 76 patients without multidose drug dispensing. In 16 (31%) out of these cases, medication lists were updated in the primary care medical records.

Patient chart entries regarding medication or medication-related follow-up were seen in the primary care medical records in 53 out of 115 (46%) cases, whereof 18/47 when the discharge summary was and 35/68 when the discharge was not received by primary care (information transfer on the hospital stay sometimes occurred via other hospital documents).

### Electronic survey

The survey was answered by 107 out of 151 (71%) primary care units. Out of these 107 primary care units, 22 (21%) respondents reported the discharge summary was *often* received on the day of discharge while 71 (66%) respondents indicated the discharge summary was *always*/*often* received, but later. In addition, 45 (42%) respondents reported that reason for drug changes was *never*/*seldom* present in the medication report. Medication list update and patient chart entry in the primary care medical records was *always/often* done upon receipt of the discharge summary according to 61 (57%) respondents. After drug changes during hospitalisation, 78 (73%) respondents indicated the treatment would *always/often* be followed-up by the doctor in charge if needed. A total of 64 (60%) respondents answered that they had discussed and reached consensus on how to handle the received discharge summary, while only 31 (29%) had prepared local written instructions for this. The discharge summary was *always/often* of great help for follow-up of the patient’s medication according to 93 (87%) respondents. Full results are presented in Table 1.

**Table 1:** Electronic survey answers on content, transfer and use of the discharge summary from 107 primary care units

	Never	Seldom	Often	Always
The discharge summary is received on the day the patient is discharged.	32 (29%)	53 (50%)	22 (21%)	0
The discharge summary is received, but not on the day the patient is discharged.	3 (3%)	33 (31%)	66 (61.5%)	5 (4.5%)
The discharge summary is not received, but the medical report is instead incorporated into the medical case history.	10 (9%)	49 (46%)	40 (37%)	8 (8%)
The information in the medical report is clearly written.	0	24 (23%)	71 (66%)	12 (11%)
The information in the medical report is reliable	0	18 (17%)	78 (73%)	11 (10%)
The reason for any drug change is indicated in the medication report.	1 (1%)	44 (41%)	52 (49%)	10 (9%)
The information in the medication list is reliable	0	22 (21%)	74 (69%)	11(10%)
Drug indication is indicated in the medication list.	1 (1%)	32 (30%)	63 (59%)	11 (10%)
The doctor in charge checks the medication list and the medical report to urgently detect any uncertainties/errors.	0	13 (12%)	63 (59%)	31 (29%)
If any uncertainty/error is detected, it is followed up by the doctor in charge.	0	14 (13%)	47 (44%)	46 (43%)
The medication list is updated and changes documented as patient chart entries when the discharge summary is received.	11 (10%)	35 (33%)	44 (41%)	17 (16%)
The medication list is not updated nor changes documented as patient chart entries until the patients next planned contact.	0	34 (32%)	53 (49%)	20 (19%)
After drug changes during hospitalisation, treatment is followed up by the doctor in charge if needed.	1 (1%)	28 (26%)	49 (46%)	29 (27%)
The discharge summary is of great help for follow-up of the patient’s medication treatment after the hospital stay.	1 (1%)	13 (12%)	39 (36%)	54 (51%)

#### Free text comments

Three major categories emerged in the summative content analysis of the free text comments: ‘Inaccuracies regarding medication, medication lists and the discharge summary’, ‘Care transition information transfer and follow-up’ and ‘How to handle the discharge summary´.

#### Inaccuracies regarding medication, medication lists and the discharge summary

Problems due to separate electronic medical records in primary care and the hospitals were pointed out. For instance, the hospital medical records (not accessible to GPs in general) could be referred to for actual medication lists, although they were not visible for GPs. A common medication list for hospital and primary care was requested. In the discharge summary, information about whether prescriptions were made at the hospital could sometimes be missing. Shortcomings in managing patients with multidose drug dispensing were also noted. It was also suggested that medication lists could already be incorrect upon a patient’s admission to hospital.

#### Care transition information transfer and follow-up

The need of a formal referral for follow-up was repeatedly noted, partly due to heavy work burden in primary care. A few respondents commented that the discharge summaries were often late or missing.

#### How to handle the discharge summary

Lack of time was pointed out as a negative factor regarding updating medication lists. A handful of respondents described local instructions on how to handle the received discharge summary to be imminent.

## Discussion

Less than half of the discharge summaries for elderly patients with many drugs were received, although they are meant to inform the patient as well as the next caregiver about drug changes made during hospital admissions. In the survey, more than one-third reported the discharge summary was never/seldom received, regardless of the time span. Furthermore, almost two-thirds in the survey indicated that they always/often updated medication list/made patient chart entries upon receipt of the discharge information. However, the medical record investigation showed that after drug changes, only one third of medication lists were updated in primary care medical records after two weeks. Patient chart entries regarding medication or its follow-up were only seen in primary care medical records in every other case.

The average age of patients in this study was 86 years old and used five or more drugs. People of this age are vulnerable and often use many drugs [7, 8], with an accordingly high risk of adverse drug reactions and hospitalisation [9, 11]. Drug-related problems are common after hospital discharge [23], and drug-related readmissions are frequent [24]. A discharge summary with a medication report improves medication use [13, 14, 16, 23]. However, to fully exploit its potential the discharge summary must be adequately transferred and received, which was seen in only about 40% of cases in this study. Poor communication and information transfer in care transitions and being perceived negatively by the GPs and contributing to medication errors have also been shown previously [25–28]. In a Swedish National board of health and welfare survey [7], only around half of the primary care units received information on medication initiated during hospitalisation, more commonly through medical case histories than medication reports [7]. Information transfer often took at least a week, sometimes even more than two weeks [7]. As expected, this study had a larger proportion of transfers since inclusion was actual discharge summaries. Nevertheless, the transfer of discharge summaries was still inadequate. Furthermore, the discharge summary should also be immediately transferred to the next caregiver. Thus, although a prolonged time period might have allowed for a greater number of discharge summaries being found, we consider this less likely. Although some studies show no difference in GPs satisfaction with electronic discharge summaries [29], others clearly demonstrate improved satisfaction regarding both quality and timeliness [30–32]. Even electronically produced discharge summaries may still be manually transferred. As in our study, the electronic discharge summaries in the study by Alderton and Callen were printed and mailed to the GP [32]. However, electronic transfer was called for, deemed desirable if doable [32]. While electronic preparation may result in improved discharge summaries, it still relies on the information derived from the electronic medical records being adequate. However, increased electronic management may help improve information transfer in care transitions. A secure way of transferring the discharge summaries electronically may prevent delays in their delivery to primary care. An electronic system may also enable security check-points such as automated reminders to hospital physicians to write and send the discharge summary, as well as compulsory receipts from primary care upon receiving the discharge summaries.

In this study, medication lists were not satisfyingly updated after drug changes, although the survey respondents reported updating more frequently. Similar to our results, a recent study from the Netherlands showed one third of in-hospital prescription changes was not or incorrectly documented in the primary care medical record [33], which is in line with a previous study on post-discharge medication-related information [34]. According to previous Swedish and American studies, discrepancies between the patients’ intended medication regimen and the medication list in primary care are common [35–37], even with a common medical record [35, 36], and the need for great improvement regarding updated medication lists was noted [38]. An accurate medication list is essential to assess the patient’s symptoms as well as the risks and effects of treatment [28]. Failure to update the medication list could possibly result in medication errors. A large share of medication errors is potentially harmful [39, 40] and may lead to preventable adverse drug events [41]. A poorly updated medication list might also partly result from the well-known occurrence of medication errors in discharge summaries [42] and a lack of important drug-related information [26, 34, 43]. Although many survey respondents viewed the medication report as reliable and clear and the discharge summary of great help for medication follow-up, many had experienced lack of reason for drug changes in the medication report thus possibly affecting the GPs’ attitude towards updating the medication list. Piecing the information together is also very time consuming. The type of drug that was changed may also affect the update, since drugs may be viewed as being of different importance. However, although not examined in our study, a previous study of discrepancies in the medication list noted cardiovascular drugs as those that are most commonly affected [35], which are likely often of importance. Regardless, updating the medication list is an essential responsibility for any physician [44].

Making a patient chart entry and updating the medication list might be considered unnecessary; since the discharge summary is scanned into the primary care medical records. However, this means uncertainty concerning which list that is correct. Since clear routines are lacking it is likely up to the individual doctor if and when a patient chart entry is made, which is a national problem [7].

Routines for content and transfer of discharge summaries may reduce care transition medication errors [7, 28]. Primary care confirmation of the information transfer before taking over responsibility for the patient has been suggested [45]. No current common routine on transfer and use of the discharge summaries exists in primary care in Sweden, merely general and guiding principles regarding medication management [46] as in some other countries [47, 48]*.* Although many of the survey respondents had discussed the topic, few had written a local routine. This may affect its use, i.e. updating the medication list and planning any follow-up.

Further, almost 30% reported never/seldom following up drug changes even if needed, which risks delayed detection of side-effects and other drug-related problems. The reasons behind abstaining from follow-up are not known. However, in the survey free text comments, the need for a formal referral for follow-up as well as lack of time to use the discharge summary was noted. Noting but not actively following up drug changes as well as insufficient time to update medication lists implies a risk of medication errors and drug-related problems. Targeting this may increase medication safety and reduce costs. The possibility of errors on admission was also highlighted. Keeping primary care medication lists accurate counteracts this. A common medication list for hospital and primary care was also proposed in the survey free text comments. Even though a common list could increase medication safety, this may fail due to unsatisfactory maintenance and frequent errors [35, 38]. In addition, hoping for a future common list does not eliminate the responsibility to keep current medication lists accurate. An inaccurate list is unreliable and unusable as well as potentially risky for the patient.

The strengths of this study are the two perspectives on studying information transfer, where the medical records review was complemented by the survey. Moreover, this study has a primary care focus on information transfer; an area that is not well explored in Sweden.

A possible limitation of this study may be the timeframe; the time period of two weeks may have contributed to the low share of updated medication lists and patient chart entries in the primary care medical records. However, despite possible delayed medical secretary writing and physicians’ heavy work burden, follow-up of these fragile patients should be initiated within this timeframe [8, 9]. Still, it is possible some GPs update the medication list at a later point in time, for example the patient’s next visit, which may affect medical safety. Furthermore, opinions of survey non-respondents (44 primary care units) are not known. It was advised to answer the survey in consultation with the chief physician. Hence, opinions of a single GP may be represented rather than those of all colleagues. As previously noted, GPs are often individualists that work in their own way, without the management leading the medical work [38]. It is possible the non-respondents would report updating medication lists and making patient chart entries to a lower extent. However, the survey was not primarily sent to the physicians but the head of the primary care unit. Further, the survey examined their views of their actions, while the first part of the study examined the actual share of updated medication lists and included all primary care units receiving discharge summaries during the study period, regardless of whether they later answered the survey or not. Nevertheless, web surveys are commonly associated with low response rates, especially physician surveys [49], hence the response rate in our study is acceptable. Further, the survey reflected all discharge summaries while the review of the primary care medical records focused on elderly patients with many drugs. However, differing views on information transfer due to patient type was not expected.

### Future research

Further improvement could result from electronic transfer of the discharge summary. Enhanced communication, possibly electronic, between hospital and primary care physicians before hospital discharge regarding for example medication changes as well as follow-up plans could also be beneficial. Also, efforts to improve information transfer at all stages, especially for elderly who are particularly vulnerable in care transitions, could be of value, such as adequate time being allowed for writing and receiving these discharge summaries. Although there are no plans for further follow-up, conducting a study with an extended time frame could be of value. Obstacles and possibilities for improving the transfer and use of discharge summaries need to be further explored. Could inadequate use be due to poor quality of the discharge summary? In addition, what information the Swedish GPs want in the discharge summary is not known, and further studies to explore their perceptions and experiences of the discharge summary could be of interest. Further, to examine whether a poorly updated medication list leads to medication errors and its possible consequences would be of value.

## Conclusions

Information transfer was insufficient when elderly patients were discharged from hospital to primary care. Many discharge summaries were lost, an unsatisfactory proportion of medication lists were updated and patient chart entries were not always seen, although stated being done more often than was actually noted. Even though discharge summaries were considered clear and reliable, important drug-related information was often noted as missing. The shortcomings noted in this study imply a risk of medication errors and drug-related problems in care transitions.

## Abbreviation

GP: General practitioner
